# Mindfulness-based treatment of addiction: current state of the field and envisioning the next wave of research

**DOI:** 10.1186/s13722-018-0115-3

**Published:** 2018-04-18

**Authors:** Eric L. Garland, Matthew O. Howard

**Affiliations:** 1 Center on Mindfulness and Integrative Health Intervention Development, University of Utah, 395 South, 1500 East, Salt Lake City, UT 84112 USA; 2 University of North Carolina at Chapel Hill, Chapel Hill, USA

**Keywords:** Automaticity, Addiction, Dissemination, Dose–response, Mindfulness, Review, Reward, SMART

## Abstract

Contemporary advances in addiction neuroscience have paralleled increasing interest in the ancient mental training practice of mindfulness meditation as a potential therapy for addiction. In the past decade, mindfulness-based interventions (MBIs) have been studied as a treatment for an array addictive behaviors, including drinking, smoking, opioid misuse, and use of illicit substances like cocaine and heroin. This article reviews current research evaluating MBIs as a treatment for addiction, with a focus on findings pertaining to clinical outcomes and biobehavioral mechanisms. Studies indicate that MBIs reduce substance misuse and craving by modulating cognitive, affective, and psychophysiological processes integral to self-regulation and reward processing. This integrative review provides the basis for manifold recommendations regarding the next wave of research needed to firmly establish the efficacy of MBIs and elucidate the mechanistic pathways by which these therapies ameliorate addiction. Issues pertaining to MBI treatment optimization and sequencing, dissemination and implementation, dose–response relationships, and research rigor and reproducibility are discussed.

## Background

Advances in biobehavioral science occurring over the past several decades have made significant headway in elucidating mechanisms that undergird addictive behavior. This large body of research suggests that addiction is best regarded as a cycle of compulsive substance use subserved by dysregulation in neural circuitry governing motivation and hedonic experience, habit behavior, and executive function [1]. Though findings from the basic science of addiction have yielded novel treatment targets that may inform the development of promising pharmacotherapies, the behavioral treatment development process often lags behind the ever-accelerating pace of mechanistic discovery. In that regard, the mainstays of behavioral addictions treatment, cognitive-behavioral therapy and motivational interviewing, were developed decades ago and prior to the current understanding of addiction as informed by neuroscience. Yet, to the extent that behavioral therapies target dysregulated neurocognitive processes underlying addiction, they may hold promise as effective treatments for persons suffering from addictive disorders.

Contemporary developments in addiction neuroscience have been paralleled by increasing interest in the age-old mental training practice of mindfulness meditation as a potential treatment for addictive behavior. This interest was sparked by the successful integration of mindfulness techniques into secularlized behavioral intervention programs, including Mindfulness-Based Stress Reduction (MBSR) [2] and Mindfulness-Based Cognitive Therapy (MBCT) [3]. Standardized mindfulness training programs were originally focused on reducing emotional distress, and indeed, for psychiatric disorders and symptoms mindfulness-based interventions (MBIs) have been shown through meta-analysis to be efficacious and comparable to other active, head-to-head treatments [4]. More recently, MBIs like Mindfulness-Based Relapse Prevention (MBRP) [5] and Mindfulness-Oriented Recovery Enhancement (MORE) [6] have been tailored to directly to address the mechanisms that undergird addiction. A growing body of controlled research studies demonstrates that MBIs may produce significant clinical benefits for users of a panoply of addictive substances, including alcohol, cocaine, nicotine, and opioids. The aims of this report were to operationalize the construct of mindfulness with respect to therapeutic processes that mediate its potential efficacy; review the current state of research on mindfulness as a treatment for addiction; and to envision the next wave of research in this emerging and important field. With regard to setting a future research agenda here we highlight issues related to: research rigor and reproducibility; treatment optimization based on mechanistic discoveries; the sequencing of MBIs in multimodal treatment packages; the need to consider dose–response relationships; the translation and dissemination of MBIs into standard, community-based addiction treatment settings; and the possibility of construing mindfulness as an integral component of a recovery-oriented lifestyle rather than a time-limited treatment.

## Mindfulness as a means of targeting mechanisms of addiction

### Mindfulness operationalized

Derived from ancient Indo-Sino-Tibetan contemplative practices and philosophies concerning the cultivation of awareness, the construct of mindfulness has been alternately operationalized as a state, trait, and practice in the modern scientific literature. MBIs provide training in practices designed to evoke the *state of mindfulness*—i.e., a state of metacognitive awareness characterized by an attentive and nonjudgmental monitoring of moment-by-moment cognition, emotion, sensation, and perception without perseveration on thoughts of past and future. The *practice of mindfulness* has been proposed to involve two primary elements: focused attention and open monitoring [7, 8]. During the practice of focused attention, attention is concentrated on a sensory object (often the sensation of breathing, but interoceptive and proprioceptive body sensations or external visual foci can also be used) while one acknowledges and then disengages from distracting thoughts and emotions. Focused attention practices often precede the practice of open monitoring, in which one observes both the arising of mental contents as well as the field of awareness in which those contents arise [7]. Open monitoring is a metacognitive state of awareness in the sense that it involves monitoring the content of consciousness while reflecting back on the process or quality of consciousness itself. This form of mindfulness practice is thought to reduce emotional reactivity by revealing the insubstantiality and ephemerality of any particular content of consciousness. Neuropsychological models of focused attention and open monitoring have mapped these practices onto systems of interacting cognitive processes, including sustained attention, attentional re-orienting, conflict monitoring, retaining information online in working memory, inhibitory control, and emotion regulation [8]. Although focused attention and open monitoring have been distinguished in the scientific literature, in practice they are often combined, such that mindfulness practices typically begin with focused attention and then develop into open monitoring as the meditation session unfolds over time.

Frequent and regular practice (e.g., daily) of mindfulness techniques is thought to cultivate durable changes in the trait-like propensity to be mindful in everyday life (i.e., *dispositional* or *trait mindfulness*) even when one is not engaged in meditation practice [9]. This increase in trait mindfulness is theorized to occur through neurocognitive plasticity kindled by repeated activation of the state of mindfulness during recurrent mindfulness practice sessions [10]. In partial support of this hypothesis, increases in the trajectory of state mindfulness produced over time through meditation predicts increases in trait mindfulness [11], and meta-analysis demonstrates that the effects of MBIs on clinical outcomes are mediated by increases in trait mindfulness [12]. Further, meta-analysis of morphometric neuroimaging suggests that increased practice of mindfulness meditation is associated with neuroplastic changes in brain structure [13]. According to operationalizations of the construct derived from factor analytic research, dispositional or trait mindfulness is characterized by the capacity to remain nonreactive to and accepting of distressing thoughts and emotions; observe interoceptive and exteroceptive experience; discriminate emotional states; and be aware of automaticity [14]. These mindful qualities may serve as antidotes to addictive behavior; indeed, trait mindfulness, which has been correlated with enhanced cognitive control capacities [15], is significantly inversely associated with substance use [16] and craving [17], and positively associated with the ability to disengage attention and recover autonomic function following exposure to addiction-related cues [18, 19]. In contrast to trait mindfulness, which is associated with cognitive and behavioral flexibility, addiction may be characterized by *mindlessness* [20], i.e., habitual or stereotyped responses that may be executed automatically without conscious volition or strategic regard for distal consequences. In light of Tiffany’s classic description of addiction as the product of automaticity [21], mindfulness of one’s automatized behavioral and emotional reactions may allow for greater self-regulation of habitual addictive behavior. Thus, mindfulness practice may evoke the state of mindfulness that accrues with each meditation practice session into a durable propensity to exhibit the trait of mindfulness in everyday life, thereby suffering as a buffer against addictive behavior.

### Mindfulness-based interventions for addiction

The most prominent MBIs (i.e., MBRP, MORE, mindfulness training for smokers) for addiction were modeled after the first generation of mindfulness-based therapies like MBSR and MBCT in terms of their structure and format. MBIs for addiction tend to be multi-week interventions (approximately 8 weeks in duration) usually delivered in a group therapy format. Each week, participants are guided by a trained clinician in various mindfulness practices, including mindful breathing and body scan meditations. These in-session mindfulness practices are debriefed during a subsequent group process, after which new psychoeducational material is typically presented. Sessions often involve experiential exercises to reinforce the mindfulness principles that had been introduced didactically. Participants are given therapeutic homework, consisting of formal and informal mindfulness practices as well as assignments to self-monitor symptoms like craving and negative affect. Extant MBIs for addiction differ from one another in terms of the types of mindfulness practices taught, the style in which these practices are delivered and debriefed (e.g., MBRP uses open, non-directive inquiry whereas MORE employs a directive approach with a high degree of positive reinforcement), the length of at home mindfulness practice sessions, and the specific psychoeducational content delivered.

MBIs for addiction are usually tailored to address pathogenic mechanisms implicated in addiction by targeting mindfulness techniques to addictive behaviors (e.g., mindfulness of craving) and by discussing the application of mindfulness skills to cope with addiction in everyday life. For instance, MORE participants are guided to engage in the “chocolate exercise”— an experiential mindfulness practice designed to increase awareness of automaticity and craving [6]. During this exercise, participants are instructed to hold a piece of chocolate close to their nose and lips and become mindful of the arising of craving as they refrain from eating the chocolate. During this exercise, a comparison is made between the urge to swallow the chocolate and craving for addictive substances. Participants are then guided to adopt a metacognitive stance toward their experience and deconstruct the craving into its constituent sensory, affective, and cognitive components, noticing how the craving subsides over time. Through this technique, clients learn to consciously and adaptively respond to the urge to use substances rather than automatically reacting to appetitive cues in maladaptive ways. Such tailoring is presumed necessary for maximizing clinical effects of MBIs as treatments for addiction, though no quantitative comparisons of tailored (e.g., MBRP) versus general (e.g., MBSR) MBIs have been conducted for individuals with substance use disorders. Comparative effectiveness research or dismantling trials are needed to determine whether such addiction-specific tailoring increases effect sizes.

### Therapeutic mechanisms of mindfulness as a treatment for addiction

In a mechanistic theoretical account of mindfulness as a treatment for addiction, Garland, Froeliger, & Howard conceptualized MBIs as means of mental training designed to exercise a number of neurocognitive processes that become dysregulated during the process of addiction [22]. Such mental training is provided by focused attention and open monitoring mindfulness practices, which in isolation and in tandem are thought to exercise processes crucial to the self-regulation of addictive behavior such as attentional re-orienting, metacognition, reappraisal, and inhibitory control [8].

**Fig. 1:**
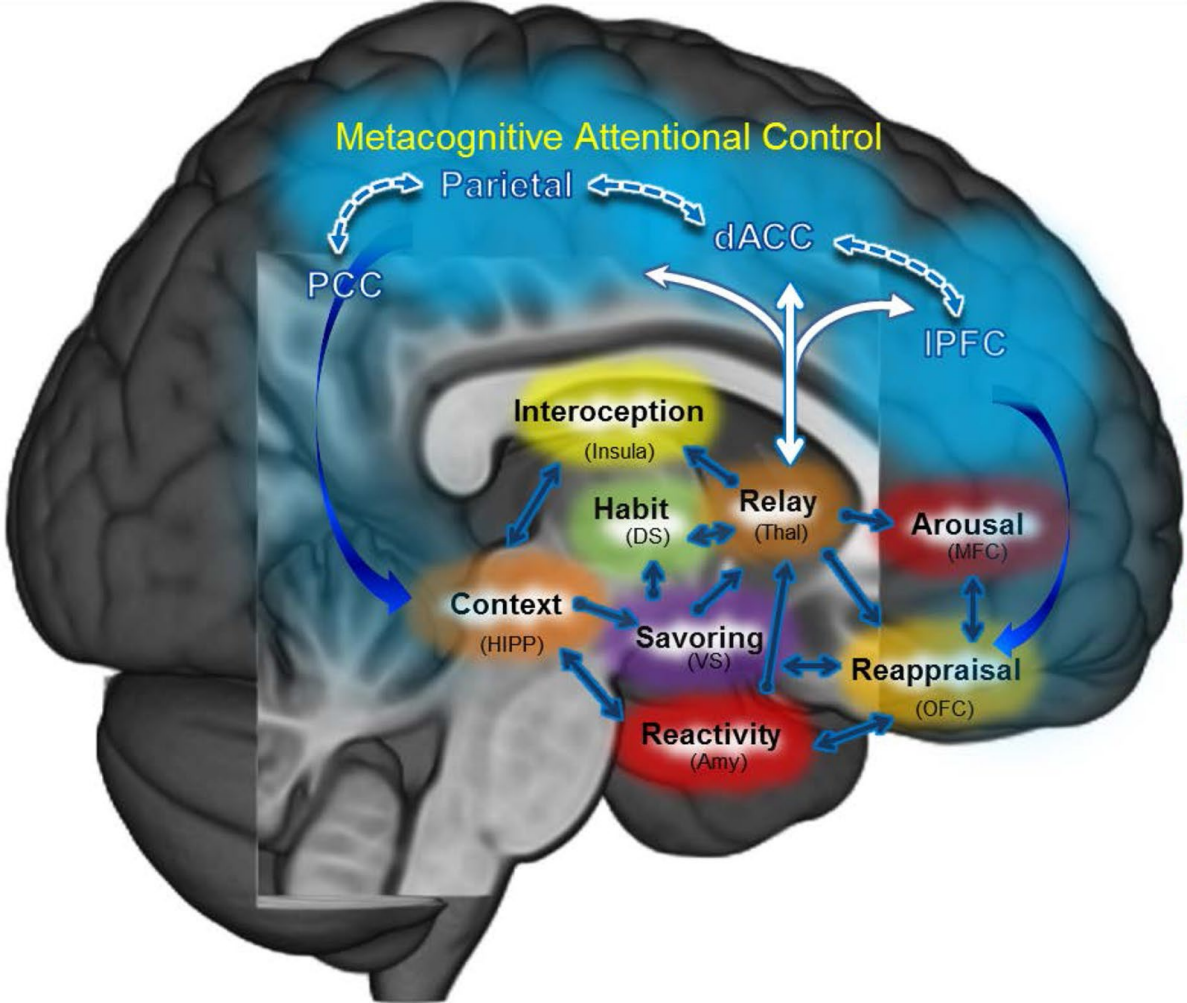
Hypothesized neural mechanisms by which mindfulness-based interventions ameliorate addictive behavior. Garland et al. [20] model of mindfulness-centered regulation posits that mindfulness-based interventions ameliorate the craving, negative affective states, and automatic habit behaviors underpinning addiction by enhancing functional connectivity (1) within a “top-down” brain network subserving metacognitive attentional (dlPFC, dACC, parietal cortex) and (2) between this metacognitive attentional control network and “bottom-up” brain structures implicated in automaticity, memory consolidation, interoception, and hedonic regulation. Enhanced functional connectivity within and between these neural circuits may allow individuals to self-regulate addictive impulses and restructure reward processes to support healthy, goal-oriented behavior. *dlPFC* dorsolateral prefrontal cortex, *dACC* dorsal anterior cingulate cortex, *PCC* posterior cingulate cortex, *DS* dorsal striatum, *VS* ventral striatum, *Thal* thalamus, *HIPP* hippocampus, *Amy* amygdala, *OFC* orbitofrontal cortex, *MFC* medial prefrontal cortex

**Fig. 2:**
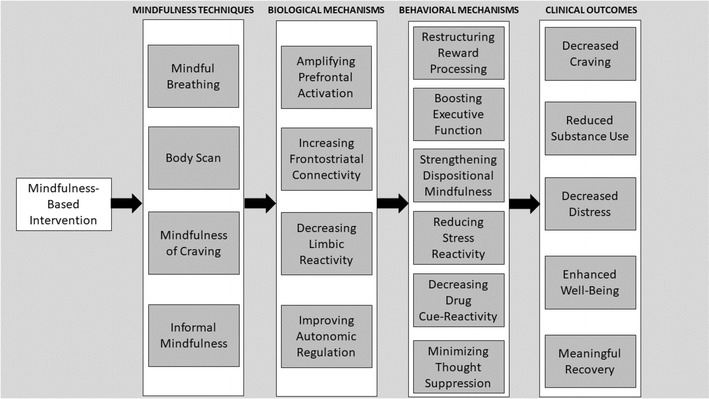
Schema detailing the effects of mindfulness-based intervention components on mechanisms and outcomes implicated in the treatment of addictive behavior

From this perspective, MBIs can been seen as behavioral strategies for strengthening the integrity of prefrontally-mediated cognitive control networks that have become atrophied by chronic drug use and hijacked by drug-related cues and cravings during the process of addiction. As adaptive cognitive control is restored through mindfulness exercises, MBIs may increase functional connectivity between these top-down prefrontal networks and bottom-up limbic-striatal brain circuitry involved in reward processing and motivated behavior [22]. Increased connectivity between top-down and bottom-up brain networks implicated in addiction (e.g., frontostriatal circuitry) may provide the physiological substrate through which mindfulness de-automatizes addictive behavior. Figure 1 depicts hypothesized neural functional mechanisms of MBIs for addiction. By augmenting the capacity of the PFC to regulate subcortical brain networks in a goal-directed manner, MBIs may strengthen a domain general neurocognitive resource that can be used to modulate a variety of mechanisms implicated in addiction, including reward processing, cue-reactivity, stress reactivity, etc. These hypothetical behavioral mechanisms are depicted in Fig. 2, and evidence for these mechanisms is reviewed below.

#### Restructuring reward

Through mechanistic effects of mindfulness-induced enhancements in functional connectivity between top-down and bottom-up brain circuitry, MBIs may reverse the allostatic process by which normal reward learning is usurped by addictive substances. In that regard, Garland recently advanced a novel hypothesis concerning the therapeutic mechanisms of mindfulness for treatment of addiction: *the restructuring reward hypothesis* [23]. The restructuring reward hypothesis states that mindfulness may reduce addictive behavior by shifting the relative salience of drug and natural rewards from valuation of drug-related reward back to valuation of natural rewards that were salient before the development of addiction. Though not the explicit aim of most MBIs, by virtue of their effects on enhancing attention regulation and positive affect, mindfulness training might nonetheless increase pleasure from perceptual and sensorimotor experiences in a fashion similar to sensate-focus techniques [24] and promote positive emotion regulation by amplifying selective attentional processes [25]. Indeed, brief mindfulness practice while eating increased ratings of subsequent food liking and enjoyment [26, 27], and 8-weeks of mindfulness training increased the experience of reward derived from pleasant daily life activities [28]. This application of mindfulness as a means of appreciating and focusing on natural rewards is termed *savoring* [29].

Though the aforementioned studies were not directly concerned with substance use disorder treatment, a mounting body of evidence supports the *restructuring reward hypothesis* and suggests that increasing natural reward processing through MBIs might reduce craving and addictive behavior. In mechanistic analyses from a RCT of MORE as a treatment for prescription opioid misuse among chronic pain patients, MORE produced significant increases in cardiac-autonomic and electrocortical responses to natural reward stimuli that were, in turn, associated with decreases in opioid craving [30, 31], suggesting that MBI may restructure reward processing. Further, recent analyses indicate that MORE enhances autonomic responses to natural reward cues relative to opioid cues, and increases in relative physiologic responsiveness to natural versus opioid-related reward significantly predicted reduced opioid misuse 3 months later [32]. These psychophysiological findings converged with ecological momentary assessment data collected during this trial, which indicated that MORE significantly increased the trajectory of positive affect from moment-to-moment which in turn predicted decreased opioid misuse following treatment [33].

These findings supporting of the *restructuring reward hypothesis* were paralleled by preliminary functional magnetic resonance imaging (fMRI) evidence of the effects of MORE on nicotine dependent smokers. In a pilot study of MORE as a smoking cessation intervention [34], smokers viewed cigarette images during a cue-reactivity task, and then in a separate positive emotion regulation task, either viewed or savored images representing natural rewards. Relative to a time matched comparison group, participants in MORE exhibited significant pre-post treatment reductions in ventral striatal responses to cigarette cues over time, and significant increases in ventral striatum and rostral anterior cingulate cortex (rACC) during savoring of natural reward stimuli. Furthermore, resting state functional connectivity between rACC and orbitofrontal cortex (OFC) significantly increased in the MORE group relative to the comparison group. These increases in functional connectivity, striatal, and rACC savoring responses significantly predicted increases in positive affect and reductions in the quantity of cigarettes smoked over the course of treatment with MORE, suggesting that mindfulness training may treat addiction by restructuring function of brain reward circuitry. To be clear, MORE provides integrated training in mindfulness, reappraisal, and savoring skills, and therefore other MBIs may or may not exert similar effects on restructuring the relative salience of natural and drug-related reward. However, other potential mechanisms of mindfulness as a treatment for addiction have been identified in the literature and are discussed below.

#### Executive functioning

By strengthening top-down cognitive control, MBIs may improve executive functions like self-control over automatic habits, decision-making, and response inhibition that are crucial to reducing drug use and maintaining abstinence. In that regard, a small quasi-experimental study of a mindfulness and goal management training intervention for polysubstance use demonstrated significant improvements in executive functioning, including working memory, selective attention/response inhibition and decision-making skills following mindfulness training relative to treatment as usual [35]. A subsequent pilot RCT with a sample of polysubstance users replicated these effects of combined goal management and mindfulness training in laboratory-based tasks and ecologically valid measures of decision-making [36]. Similarly, in a full-scale clinical trial, mindfulness-based addiction treatment significantly improved smoking abstinence by decreasing concentration difficulties [37]. In addition, there is some evidence that MBIs for addiction increase activation in brain regions implicated in self-regulatory executive functions: a small RCT showed that 2 weeks of mindfulness training was associated with significant reductions in smoking coupled with increased resting state activity in the ACC and mPFC [38]. Such increased prefrontal activation might facilitate mindfulness-induced deautomatization of addictive responses.

#### Dispositional mindfulness

MBIs might also reduce addictive behavior by strengthening facets of dispositional/trait mindfulness. In a RCT of MBRP among a heterogenous sample of individuals with various substance use disorders, increases in dispositional mindfulness facets like acceptance, awareness, and nonjudgment significantly mediated the effect of MBRP on decreasing craving following treatment [39]. Similarly, in a large cluster RCT of MORE versus CBT or TAU, increases in dispositional mindfulness significantly mediated the effect of MORE on reducing craving following treatment [40]. Finally, MORE significantly increased the mindfulness facet of nonreactivity which, in turn, predicted decreases in prescription opioid misuse [41].

#### Stress reactivity and stress recovery

Given known linkages between stress and addiction [42, 43], MBIs might ameliorate addictive behavior by attenuating stress reactivity and augmenting stress recovery. Several studies of MBIs as treatments for addiction have employed measures of heart rate variability (HRV), the beat-to-beat variation in heart rate driven by the parasympathetic nervous system [44], as an index of the capacity to regulate physiological reactivity and recovery from stress. In a sample of individuals receiving treatment for substance use disorders, relative to a control group and treatment-as-usual, MBRP was associated with significantly greater increases in tonic HRV and phasic HRV responses to stress [45]. Similarly, a mindfulness training intervention based on MBRP for individuals with alcohol and/or cocaine use disorders was associated with significantly attenuated sympathovagal HRV ratio during stress exposure [46]. Among nicotine-deprived smokers, brief mindfulness training was associated with significantly greater HRV during stress exposure than a control condition [47]. In a pilot RCT, compared to an active control group, participation in MORE was associated with significantly greater HRV recovery from stress-primed alcohol cues that were coupled with significantly greater reductions in cue-induced distress over the recovery period [48]. With regard to stress biomarker measures, one study found that mindfulness training for smokers was associated with significant within-group decreases in hair cortisol measures [49], and an nonexperimental study of MBSR found significant within-group decreases in awakening salivary cortisol levels among participants receiving inpatient treatment for substance use disorders [50]. To date, only one study has examined brain mechanisms of the stress regulatory effects of MBIs: in a RCT, an 8-week MBI for smoking cessation was associated with significantly less amygdala and insula activation during stress exposure relative to an active control condition, and reduced activity in these brain regions was associated with decreased smoking by follow-up [51].

#### Craving and cue-reactivity

MBIs may reduce addictive behavior by decreasing subjective craving and attentional and physiological indices of drug cue-reactivity. In addition to the aforementioned smoking cessation study in which MORE reduced striatal responses to cigarette cues [34], decreased cue-reactivity was observed in an RCT in which MORE was shown to significantly reduce attentional bias toward opioid cues [52] and decrease subjective craving responses during an opioid cue-reactivity protocol [30]. Similarly, in a lab-based brief mindfulness induction, mindful attention to smoking cues significantly reduced craving coupled with decreased activation in a craving-related region of the subgenual ACC [53]. These lab-based assessments of craving as a mediator of MBI effects have been corroborated by clinical research: in a large clinical trial, the effects of mindfulness-based addiction treatment on smoking abstinence were mediated by decreased craving [37]. Similarly, a study of mindfulness training for smokers found that mindfulness significantly reduced post-quit smoking urge ratings that were significantly associated with smoking abstinence [54]. In addition to targeting craving, mindfulness training aims to reduce cognitive, affective, and behavioral reactivity. In the context of addiction, substance use is often a reaction to increases in negative affect and craving. Thus, MBIs may undo this reaction by decoupling affective response and craving from substance use. For example, brief instruction in mindfulness as a means of coping with urges to smoke attenuated the association between negative affect and smoking urges [55]. With regard to longer MBI programs, MBRP has been shown to decouple associations between depressed mood, craving, and substance use; MBRP significantly reduced the association between postintervention depressive symptoms and craving 2 months following treatment, which in turn predicted reduced substance use at 4-months follow-up [41, 56]. A mindfulness-based smoking cessation intervention derived from MBRP significantly reduced the association between craving and cigarette smoking [57]. In the same vein, MORE significantly attenuated the association between prescription opioid craving and opioid misuse.

#### Thought suppression

Finally, given that suppression of addictive urges exhausts autonomic resources for self-control [58] and paradoxically amplifies craving [59], MBIs may reduce addictive behavior by providing an effective alternative to thought suppression. In support of this notion, a quasi-experimental study of incarcerated substance users found that decreases in thought suppression mediated the effect of mindfulness training (i.e., Vipassana meditation) on reducing alcohol use [60]. Similarly, in a pilot RCT of alcohol dependent inpatients, MORE was shown to significantly reduce thought suppression which was in turn associated with reductions in alcohol attentional bias [48].

Though mechanistic research on MBIs has begun to amass, there are few psychophysiological and neuroimaging studies of MBIs as a treatment for addiction. Thus little data exists to either support or refute the neural mechanistic models proposed in this section. Clearly, more research is needed in this area.

## Current state of the field: a review of clinical outcomes of mindfulness-based treatments for addiction

A considerable body of findings has amassed supporting the capacity of MBIs to reduce substance use and attenuate factors promoting substance use, such as craving and stress. Over the past decade, multiple systematic reviews have been conducted to identify the effects of MBIs on addictive behaviors, and have found accumulated evidence for the positive effects of MBIs [61–63]. More recently, a meta-analysis focused on the broad clinical efficacy of MBIs for a range pf psychiatric disorders conducted subgroup analyses to examine the effects of MBIs on addiction/smoking and found MBIs to be superior to active control conditions and comparable to other evidence-based treatments [4]. In the only published meta-analysis solely focused on MBIs for substance misuse, Li, Howard, Garland, McGovern, and Lazar [64] identified 34 randomized controlled trials differing in terms of the types of MBI and comparison groups contrasted, sample demographics, and measures of outcomes and other constructs. Despite the notable methodological heterogeneity of these investigations, the authors concluded that “virtually all studies found that mindfulness treatments were associated with superior treatment outcomes at posttreatment and follow-up assessments compared to comparison conditions” (p. 69). Effects (Cohen’s d/odds ratios) ranged from moderate-to-large across the synthesized effect sizes computed for studies within the substance use (*d *= 0.33, − 0.49 to 0.17, p < 0.05), cigarette smoking (OR = 1.76, 0.99–3.15, p = 0.056), craving (*d *= 0.68, − 1.11 to − 0.025, p < 0.01), and stress (*d *= 1.12, − 2.24 to –0.01, p < 0.05) domains.

With regard to secondary or mechanistic outcomes, as expected, MBIs produced significant increases on the Five Factor Mindfulness Questionnaires in all eight studies that used this measure (*d *= 0.62, − 0.02 to 1.26, p = 0.057). In individual studies, MBIs produced a host of other significant salutary effects including increases in emotion regulation [41, 54], substance-related self-efficacy [65, 66], and positive emotions [33], as well as decreases in attentional bias [52, 66, 67], addictive automaticity [66], dysphoric affect [40, 66], and pain severity and related functional interference in patients with chronic pain [41]. Several studies reported positive associations between the degree to which participants engaged in mindfulness homework exercises and changes in cigarette, marijuana, and alcohol use posttreatment (e.g., [68–70]).

Of the 34 RCTs reviewed in this meta-analysis, ten used treatment-as-usual comparison groups, whereas two used inert comparison groups, sixteen employed an alternative psychotherapeutic treatment (typically matched to the MBI group vis-à-vis intensity, duration, and format), and six examined brief mindfulness treatments compared to alternative therapies in laboratory settings. Twenty-eight of the reports presented the first published findings from the related study and six reports presented results of secondary analyses. Any given study could contribute findings only once to meta-analyses conducted within outcome domains. The adequacy of randomization was examined in all studies and analysis of covariance and linear mixed modeling were often used to control for any remaining pretreatment differences. Nearly half of the studies had samples sizes less than fifty. Many studies had high attrition rates at posttreatment and subsequent follow-ups. Most of the 34 studies reviewed relied extensively on self-report measures of substance use and other constructs. All RCTs examined were single-site studies. The most common methodological limitations were failure to interview collateral informants regarding study participants’ substance use behaviors at posttreatment and follow-up and to employ posttreatment and follow-up interviewers who were blind to participants’ treatment assignments. Fewer than half of the RCTs employed objective verification of participants’ self-reported substance use, such as urinanalysis.

Subgroup analyses within outcome domains indicated that MORE treatment was associated with larger effects than other MBIs for substance use, craving, stress, and mindfulness measures [64]. Studies comprised entirely of men also reported larger effects for MBIs compared to studies with samples comprised only of women or those with mixed gender samples across measures of craving, stress, and mindfulness.

Li et al. [64] also reported findings from a random effects meta-regression analysis examining effects of MBI type, primary type of substance misused, study sample size, sample age and gender distributions, type of comparison group, treatment dosage in hours, and study methodological rigor on effect sizes by domain. Results indicated that studies with samples of only men experienced larger reductions in levels of craving and stress, and significantly larger increases in levels of mindfulness, compared to studies with samples comprised only of women or studies with samples comprised of women and men. Although the authors did not include a formal search for “gray literature” related to MBI treatment of substance misuse, they noted that funnel plots and Egger’s test analyses suggested that their findings were not likely due to publication bias.

Randomized controlled trials suggest that MBIs are a promising treatment for substance misuse and exert their effects via increases in levels of mindfulness across a wide array of substance-misusing behaviors and clinical populations. Future research should employ larger samples, longitudinal designs with follow-up periods of at least 1-year, manualized interventions with treatment fidelity assessment, intent-to-treat analyses, and probability sampling designs allowing generalizability to specific clinical and general populations.

## Laying out a research agenda

### Research rigor and reproducibility

MBIs are promising treatments for addiction. Results from rigorous, full-scale RCTs indicate that MBIs can produce short and long-term reductions in craving and addictive behavior. At this juncture in the development of the field, additional Stage III and IV clinical trials (for a review of the NIH Stage Model, see [71]) are needed to replicate these promising findings via gold-standard research design features including the use of active control conditions, detailed fidelity monitoring procedures, and triangulation of self-reported outcomes with biochemical verification of drug use and blinded clinical evaluations. With additional replications of positive clinical outcomes, MBIs could rightfully be considered empirically-supported therapies for addictive behaviors. Conversely, replication failures could indicate the need to “return to the drawing board” and engage in treatment development research to optimize the next generation of MBIs as interventions for addiction. Thus, in the lifespan of this nascent field, it is now an opportune moment to answer definitively the question “Are MBIs efficacious and comparatively effective treatments for addiction?”

Assuming an affirmative answer to the aforementioned question, studies should then aim to address research questions pertaining to *mediation* (“How do MBIs improve addiction-related outcomes?”) and *moderation* (“For whom do MBIs work most optimally to improve addiction-related outcomes?”). As discussed in “Mindfulness as a means of targeting mechanisms of addiction” section, a corpus of research has begun to amass on the mediators of MBI effects on addiction. In contrast, there is very little research on moderators of MBIs. The only study of MBI moderators for addiction outcomes is a secondary analysis of data from two RCTs of MBRP, which found that patients with greater substance use disorder severity and more affective symptoms received significantly greater benefit from mindfulness training than patients with low levels of substance use and affective symptoms [72].

A number of additional research questions remain unanswered. Here we lay out an agenda for the next wave of research in the field.

### Elucidating the neurobiological mechanisms of mindfulness as a treatment for addiction

Little is known about the neurobiological mechanisms of mindfulness as a treatment for addiction. Though various conceptual models have been advanced [22, 23, 73], few tests of these specific neural hypotheses have been conducted. Adequately powered, randomized fMRI studies are needed to test basic mechanistic assumptions long held in the field. For instance, do MBIs decrease addictive behavior by strengthening inhibitory control via activation of top-down neural circuitry? Do MBIs decrease addictive behavior by reducing activation of bottom-up neural circuitry to drug cues? Similarly, functional neuroimaging methods are needed to test novel hypotheses, such as the *restructuring reward hypothesis* (“Do MBIs restructure the relative responsiveness to drug and natural rewards by increasing functional connectivity between top-down and bottom-up neural circuits?”). Furthermore, molecular neuroimaging (e.g., positron emission tomography; PET) is needed to understand effects of MBIs on neurotransmitters and neuropeptides implicated in addictive behavior like dopamine, endogenous opioids, γ-aminobutyric acid (GABA), and endocannabinoids. Finally, dynamic effects of mindfulness practice on addictive responses are unknown, and could be elucidated through functional neuroimaging techniques with high temporal resolution like electroencephalography (EEG) or magnetoencephalography (MEG). Such methods could answer other pertinent questions. For instance, does the acute state of mindfulness attenuate initial attentional orienting to drug cues? Or, does mindfulness facilitate attentional disengagement and recovery from drug cue-exposure? These questions can be answered by investigating how mindfulness training influences the time course of neural responses to drug cues.

Although understanding treatment mechanism is not necessary to establish a given treatment modality as an empirically supported intervention, understanding the mechanisms of mindfulness can inform the refinement of MBIs to yield larger effect sizes and produce additional therapeutic benefits. A case in point is MORE, which was refined based on mechanistic discoveries. Following the first trial of MORE, it was found that mindfulness reduces pain severity by fostering a shift from affective to sensory processing of pain as innocuous sensory information [74]. As a result of this discovery, when MORE was optimized as a treatment for prescription opioid misuse among chronic pain patients, the intervention was modified to include a “mindfulness of pain” technique that involved using mindfulness to deconstruct pain into its sensorial subcomponents and disentangle sensation from its affective overlay. Similarly, evidence that increasing physiological responsiveness to natural rewards via mindful savoring predicts decreased prescription opioid misuse [75] and craving [30] has led to an enriched emphasis on mindful savoring practice in the MORE intervention. It is possible that these intervention refinements may account for the changes in brain reward circuitry function observed among smokers treated with MORE [34]. As another example, recent investigation of the role of the posterior cingulate cortex in meditation experience has implicated this brain region as a target for neurofeedback interventions to potentiate the efficacy of MBIs [76], and indeed, trials of such neurofeedback-enhanced MBIs are underway (e.g., NCT02413177).

### Sequencing of mindfulness as a part of multimodal treatment packages

It is not known whether MBIs are most efficacious as standalone treatments or as a part of a more comprehensive treatment package. In many inpatient addictions treatment programs, clients receive multiple behavioral interventions (e.g., motivational enhancement therapy, cognitive-behavioral therapy, dialectical behavior therapy) during the same 30-day time frame. Further, optimal treatment sequencing has not been studied. For instance, would MBIs be more efficacious following several sessions of motivational interviewing? Given that MBIs involve mindfulness practice, and regular practice requires motivation, introducing several sessions of motivational interviewing before initiating a course of mindfulness training might increase practice engagement and thereby boost clinical outcomes. Conversely, mindfulness training might potentiate motivational enhancement therapy by increasing interoceptive awareness of adverse consequences of addictive behavior on bodily health. In a similar vein, mindfulness training might increase adherence to medication-assisted therapy (MAT) by increasing awareness of how medication adherence allays the dysphoria associated with craving and thereby potentially improves quality of life. In turn, MAT might improve adherence to MBIs by attenuating distracting withdrawal symptoms and decreasing obsessive thinking about obtaining the next drug dose, thereby freeing cognitive and motivational resources to devote to learning mindfulness skills. Psychopharmacological interventions, cognitive training via computer- or smartphone-deployed technology, neurofeedback, and neurostimulation (via transcranial magnetic stimulation or transcranial direct current stimulation) administered prior to initiating a course of MBI might also improve cognitive function to facilitate learning of mindfulness techniques, and thereby improve MBI outcomes.

Sequential, multiple assignment, randomized trials (SMART) could be used to assess the efficacy of dynamic treatment regimens, including those that are individually tailored based on decision rules that dictate how the type or dosing of treatment should change based on the specific clinical needs of the patient [77]. For instance, MBI non-responders might need a supplementary course of motivational enhancement therapy, computerized cognitive remediation, or booster sessions (see “The Need for Dose/Response Research” below) to enhance outcomes. Finally, given that many MBIs are multimodal in nature and combine various mindfulness meditation practices and psychoeducational modules, studies that employ the multiphase optimization strategy (MOST) could also be used to examine the independent and additive effects of various MBI treatment components on addictive behaviors [78]. The MOST research process could allow for resource-intensive and complex MBIs to be pared down to their most efficacious elements to maximize efficacy and efficiency by eliminating techniques that do not confer therapeutic benefits and augmenting those that do.

### The need for dose–response research

In pharmacological research, it is imperative to examine dose–response relationships to identify the optimal therapeutic dose. Dose–response curves can help to identify the dose needed to achieve a satisfactory clinical outcome while minimizing the side-effect profile of the drug. Although MBIs delivered in clinical settings appear to have few adverse effects [79], the costs and time required to deliver complex behavioral treatments like MBIs necessitate dose–response considerations to identify the minimal therapeutic dose. Null effects of MBIs observed in Stage II or III clinical trials might very well be qualified by extent of mindfulness practice, and thus mindfulness practice engagement should be tested as a treatment outcome moderator. Furthermore, responder analyses might reveal that individuals classified as non-responders are those who do not meet the minimal therapeutic dose of mindfulness skill practice whereas individuals classified as responders are those who surpass this minimal therapeutic dose of practice.

Given meta-analytic findings that extent of mindfulness practice is significantly associated with treatment outcomes [80], different doses of mindfulness practice might produce different therapeutic effect sizes or different durations of therapeutic effects for addicted populations. Most MBIs for addictive disorders (e.g., MBRP and MORE) are approximately 2 months in length given that they were modeled on the canonical 8-week structure of MBSR [81]. However, due to their clinical complexity, individuals with substance use disorders are typically excluded from participating in MBSR. Although MBIs like MORE and MBRP have produced significant reductions in addictive behaviors [64], it is plausible that to achieve full remission from moderate-to-severe substance use disorder, patients might require additional weekly treatment sessions beyond the standard 8-weeks of treatment. Moreover, following a full course of a multi-week MBI, periodic booster sessions might be needed to extend treatment benefits for the long-term. Such booster sessions could come in the form of mindfulness practice sessions (with or without group process and psychoeducational content) conducted via in-person or telemedicine formats, and their additive efficacy could be tested with SMART research designs.

### The challenge of dissemination/implementation

One of the greatest challenges confronting the movement towards evidence-based practice in addictions treatment is the research-to-practice gap: that is, empirically-supported therapies with proven efficacy as revealed by Stage II randomized clinical trials are often not successfully translated into effective clinical interventions in standard addiction practice settings [82]. Successful transfer of research to practice involves programmatic change in the form of activities including exposure, adoption, implementation, and practice of new empirically-supported approaches [83]. These activities are especially complicated in the context of MBI implementation, insofar as many common MBIs require intensive instructor training. For example, the MBSR certification process costs more than $10,000 and requires approximately 3 years to complete depending on how long it takes a prospective instructor to meet the requirements, which include personal practice and participation in multi-day meditation retreats, didactic and experiential workshop training, experience leading multiple MBSR groups, and clinical supervision [84]. Further complicating this issue, individuals without clinical licensure can be certified in MBSR, yet most addictions treatment settings require staff to be licensed healthcare professionals. In contrast, other MBIs like MORE require clinical licensure but entail a much briefer and less costly training process. It remains an open question for future research as to how much clinical training, supervision, and personal practice experience is required for effective implementation of MBIs in clinical settings. Moreover, it is not known which training formats are most effective (in person, online, role play, virtual reality, etc.) in disseminating MBIs. Issues around treatment fidelity are also crucial to successful implementation of MBIs in clinical practice. However, few fidelity measures have been validated for MBIs for addiction (for a notable exception, see 85), treatment fidelity research is time intensive, and little is known about empirical relations between clinician training format, therapist adherence/competence, and MBI treatment outcomes. Similarly, the acceptability of MBIs may influence their implementation in clinical practice settings. Factors influencing the acceptability of MBIs for the treatment of addiction are poorly understood. For instance, it is plausible that patients who initially experience mindfulness meditation as rewarding (i.e., alleviating psychological distress and generating positive sensations and emotions) or who are positively reinforced by the therapist for engaging in meditation practice may be most likely to continue to practice mindfulness skills. In contrast, patients who experience an exacerbation of aversive thoughts and feelings during meditation or who receive neutral responses from the therapist might be most likely to drop out from an MBI. Moreover, non-specific factors like therapeutic alliance, and allegiance might drive MBI acceptability, adherence, and outcome in a similar fashion to other behavioral therapies. Strategic attention to such factors might in fact boost the uptake and clinical efficacy of MBIs.

In outlining issues pertaining to advancing the clinical science of MBIs, Dimidjian and Segal highlight the tension between the need to make MBIs disseminable in the context of real-world resource constraints and complex client populations while not allowing outcomes to suffer as MBIs are scaled up in the translation to community treatment settings [86]. This is indeed a challenge, as MBIs with demonstrated efficacy in Stage II trials may fail to show effectiveness in Stage III and IV trials when delivered by community clinicians. Yet, work now needs to be done to understand the feasibility, acceptability, and impact of delivering MBIs in addiction treatment settings.

### Mindfulness as a relapse prevention strategy versus mindfulness as a vehicle for recovery

Finally, it is unknown whether mindfulness might best ameliorate addiction through participation in time-limited interventions or if mindfulness should be used daily as part of a wellness lifestyle. With regard to the latter, shifting from an addiction-oriented lifestyle to adoption of a wellness lifestyle is conceptualized as integral to the recovery model [87]. In this vein, studies should examine mindfulness not only as a technique in circumscribed interventions to prevent addiction relapse but also examine mindfulness as a long-term, sustainable health behavior that promotes addiction recovery. Pursuit of a healthy lifestyle is not something that is finalized over the course of an 8-week intervention; to the contrary, maintenance of physical health requires ongoing, regular exercise and nutritious dietary choices on a daily basis that do not exceed the caloric needs of the individual. Why then should mindfulness practice be any different? As a point of consideration, 12-Step programs encourage participation in regular meetings for the entirety of one’s life. Similarly, mindfulness might need to be practiced daily or nearly every day on an ongoing basis to achieve durable therapeutic effects and maintain addiction recovery, especially in view of the chronicity of addictive disorders.

From a neurobiological perspective, increasing grey matter density, strengthening of white matter tracts, synaptic remodeling, and other neuroplastic modifications to brain structure and function needed to undo the pathophysiology of addiction might require recurrent mindfulness practice for the long-term. From a psychological perspective, long-term mindfulness practice may be needed to induce self-referential plasticity and facilitate flexible reconfiguration of the self-schema in relation to the world [88] so as to restructure reward processes away from valuation of drug reward and towards valuation of personally meaningful pursuits and relationships [23, 29]. This latter process is consistent with the ancient soteriological intention of mindfulness as a means of reducing craving by gaining insight into the true nature of the self as impermanent and interdependent [89]—paralleling Bateson’s classical cybernetic model of addiction recovery [90].

## Conclusion

The study of mindfulness as a treatment for stress and chronic pain is more than 30 years old, and researchers have investigated mindfulness as a treatment for depression for more than two decades, yet it is only in the past 10 years that research on MBIs for addiction has proliferated. This is a young scientific field, and more research is needed to elucidate the clinical outcomes and mechanisms of this promising new treatment approach for addictive disorders. One recent meta-analysis [64] indicates that MBIs produce statistically significant effects on craving (pooled Cohen’s *d *= 0.68) and substance misuse (pooled Cohen’s *d *= 0.33), suggesting that MBIs may be efficacious treatments for addiction. Overall, a number of RCTs with active control conditions have been conducted in the past decade—a sign that the methodological rigor of this field is increasing. However, with several notable exceptions (e.g., [40, 91, 92]), few studies of MBIs for addiction have had large enough sample sizes to ensure the robustness and reproducibility of clinical outcomes. Moreover, few long-term follow-ups have been conducted to assess the durability of observed treatment effects. In addition, as indicated earlier, little is known about mediators and moderators of MBIs for addiction, although understanding how and for whom MBIs work is crucial to the overall evolution of this therapeutic approach. Lastly, research is needed to situate MBIs into treatment sequences with high external validity that adaptively address the needs of responders and non-responders in a way that can be realistically implemented in community-based treatment settings. Thus, the nascent field of mindfulness treatment for addictive behaviors remains open to rigorous, scientific exploration and in need of innovative research questions and methodologies.

Coming full circle, MBIs are some of the newest additions to the armamentarium of addictions treatment. It is perhaps no coincidence that the rise of MBIs has been co-incident with advances in the neuroscience of substance use disorders. In recognizing that addiction is, in large part, mediated by cognitive and behavioral automaticity propelled by alterations to hedonic regulatory systems in the brain, this perennial form of human suffering may be especially tractable to treatment approaches like mindfulness that enhance top-down conscious control over bottom-up automatic habits and motivational drives. Insofar as the original purpose of many mindfulness meditation practices was to extinguish craving by revealing the “middle way” between attachment to pleasure and aversion to pain, MBIs may ultimately provide a skillful means of liberating the individual from the push and pull of hedonic dysregulation underlying addiction.

## References

[1] Koob GF, Volkow ND (2016). Neurobiology of addiction: a neurocircuitry analysis. Lancet Psychiatry.

[2] Kabat-Zinn J (1990). Full catastrophe living.

[3] Segal Z, Williams JM, Teasdale JD (2002). Mindfulness-based cognitive therapy for depression.

[4] Goldberg SB, Tucker RP, Greene PA, Davidson RJ, Wampold BE, Kearney DJ (2018). Mindfulness-based interventions for psychiatric disorders: a systematic review and meta-analysis. Clin Psychol Rev.

[5] Bowen S, Chawla N, Marlatt GA (2010). Mindfulness-based relapse prevention for addictive behaviors.

[6] Garland EL (2013). Mindfulness-oriented recovery enhancement for addiction, stress, and pain.

[7] Lutz A, Slagter HA, Dunne JD, Davidson RJ (2008). Attention regulation and monitoring in meditation. Trends Cogn Sci.

[8] Vago DR, Silbersweig DA. Self-awareness, self-regulation, and self-transcendence (S-ART): a framework for understanding the neurobiological mechanisms of mindfulness. Front Hum Neurosci [Internet]. 2012 [cited 2017 Oct 8];6. https://www.ncbi.nlm.nih.gov/pmc/articles/PMC3480633/. 10.3389/fnhum.2012.00296 PMC3480633 23112770

[9] Tang Y-Y. Traits and states in mindfulness meditation. In: The neuroscience of mindfulness meditation [Internet]. Cham: Palgrave Macmillan; 2017 [cited 2017 Sep 27]. p. 29–34. https://link.springer.com/chapter/10.1007/978-3-319-46322-3_4.

[10] Tang Y-Y, Hölzel BK, Posner MI (2015). The neuroscience of mindfulness meditation. Nat Rev Neurosci.

[11] Kiken LG, Garland EL, Bluth K, Palsson OS, Gaylord SA (2015). From a state to a trait: trajectories of state mindfulness in meditation during intervention predict changes in trait mindfulness. Pers Individ Dif.

[12] Gu J, Strauss C, Bond R, Cavanagh K (2015). How do mindfulness-based cognitive therapy and mindfulness-based stress reduction improve mental health and wellbeing? A systematic review and meta-analysis of mediation studies. Clin Psychol Rev.

[13] Fox KC, Nijeboer S, Dixon ML, Floman JL, Ellamil M, Rumak SP (2014). Is meditation associated with altered brain structure? A systematic review and meta-analysis of morphometric neuroimaging in meditation practitioners. Neurosci Biobehav Rev.

[14] Baer RA, Smith GT, Hopkins J, Krietemeyer J, Toney L (2006). Using self-report assessment methods to explore facets of mindfulness. Assessment.

[15] Anicha CL, Ode S, Moeller SK, Robinson MD (2012). Toward a cognitive view of trait mindfulness: distinct cognitive skills predict Its observing and nonreactivity Facets. J Pers.

[16] Karyadi KA, VanderVeen JD, Cyders MA (2014). A meta-analysis of the relationship between trait mindfulness and substance use behaviors. Drug Alcohol Depend.

[17] Garland EL, Roberts-Lewis A, Kelley K, Tronnier C, Hanley A (2014). Cognitive and affective mechanisms linking trait mindfulness to craving among individuals in addiction recovery. Subst Use Misuse.

[18] Garland EL (2011). Trait mindfulness predicts attentional and autonomic regulation of alcohol cue-reactivity. J Psychophysiol.

[19] Garland EL, Boettiger CA, Gaylord S, Chanon VW, Howard MO (2012). Mindfulness is inversely associated with alcohol attentional bias among recovering alcohol-dependent adults. Cognit Ther Res.

[20] Langer EJ (1992). Matters of mind: mindfulness/mindlessness in perspective. Conscious Cogn.

[21] Tiffany ST (1990). A cognitive model of drug urges and drug-use behavior: role of automatic and nonautomatic processes. Psychol Rev.

[22] Garland EL, Froeliger B, Howard MO. Mindfulness training targets neurocognitive mechanisms of addiction at the attention-appraisal-emotion interface. Front Psychiatry [Internet]. 2013 [cited 2017 Sep 27];4. https://www.ncbi.nlm.nih.gov/pmc/articles/PMC3887509/. 10.3389/fpsyt.2013.00173 PMC3887509 24454293

[23] Garland EL (2016). Restructuring reward processing with mindfulness-oriented recovery enhancement: novel therapeutic mechanisms to remediate hedonic dysregulation in addiction, stress, and pain. Ann N Y Acad Sci.

[24] Masters WH, Masters VJ. Human sexual response [Internet]. Bantam Books; 1986 [cited 2017 Oct 8]. http://agris.fao.org/agris-search/search.do?recordID=US201300428002.

[25] Wadlinger HA, Isaacowitz DM (2010). Fixing our focus: training attention to regulate emotion. Soc Psychol Rev.

[26] Hong PY, Lishner DA, Han KH, Huss EA (2011). The positive impact of mindful eating on expectations of food liking. Mindfulness.

[27] Hong PY, Lishner DA, Han KH (2014). Mindfulness and eating: an experiment examining the effect of mindful raisin eating on the enjoyment of sampled food. Mindfulness.

[28] Geschwind N, Peeters F, Drukker M, van Os J, Wichers M (2011). Mindfulness training increases momentary positive emotions and reward experience in adults vulnerable to depression: a randomized controlled trial. J Consult Clin Psychol.

[29] Garland EL, Farb NA, Goldin PR, Fredrickson BL (2015). Mindfulness broadens awareness and builds eudaimonic meaning: a process model of mindful positive emotion regulation. Psychol Inq.

[30] Garland EL, Froeliger B, Howard MO (2014). Effects of mindfulness-oriented recovery enhancement on reward responsiveness and opioid cue-reactivity. Psychopharmacology.

[31] Garland EL, Froeliger B, Howard MO (2015). Neurophysiological evidence for remediation of reward processing deficits in chronic pain and opioid misuse following treatment with mindfulness-oriented recovery enhancement: exploratory ERP findings from a pilot RCT. J Behav Med.

[32] Garland EL (2015). Mindfulness-oriented recovery enhancement modulates neurocognitive mechanisms and reward system function in addiction, stress, and pain.

[33] Garland EL, Bryan CJ, Finan PH, Thomas EA, Priddy SE, Riquino MR (2017). Pain, hedonic regulation, and opioid misuse: modulation of momentary experience by mindfulness-oriented recovery enhancement in opioid-treated chronic pain patients. Drug Alcohol Depend.

[34] Froeliger B, Mathew AR, McConnell PA, Eichberg C, Saladin ME, Carpenter MJ (2017). Restructuring reward mechanisms in nicotine addiction: a pilot fMRI study of mindfulness-oriented recovery enhancement for cigarette smokers. Evid Based Complement Alternat Med.

[35] Alfonso JP, Caracuel A, Delgado-Pastor LC, Verdejo-García A (2011). Combined goal management training and mindfulness meditation improve executive functions and decision-making performance in abstinent polysubstance abusers. Drug Alcohol Depend.

[36] Valls-Serrano C, Caracuel A, Verdejo-Garcia A (2016). Goal management training and mindfulness meditation improve executive functions and transfer to ecological tasks of daily life in polysubstance users enrolled in therapeutic community treatment. Drug Alcohol Depend.

[37] Spears CA, Hedeker D, Li L, Wu C, Anderson NK, Houchins SC (2017). Mechanisms underlying mindfulness-based addiction treatment versus cognitive behavioral therapy and usual care for smoking cessation. J Consult Clin Psychol.

[38] Tang Y-Y, Tang R, Posner MI (2013). Brief meditation training induces smoking reduction. Proc Natl Acad Sci USA.

[39] Witkiewitz K, Bowen S, Douglas H, Hsu SH (2013). Mindfulness-based relapse prevention for substance craving. Addict Behav.

[40] Garland EL, Roberts-Lewis A, Tronnier CD, Graves R, Kelley K (2016). Mindfulness-oriented recovery enhancement versus CBT for co-occurring substance dependence, traumatic stress, and psychiatric disorders: proximal outcomes from a pragmatic randomized trial. Behav Res Ther.

[41] Garland EL, Manusov EG, Froeliger B, Kelly A, Williams JM, Howard MO (2014). Mindfulness-oriented recovery enhancement for chronic pain and prescription opioid misuse: results from an early-stage randomized controlled trial. J Consult Clin Psychol.

[42] Sinha R (2008). Chronic stress, drug use, and vulnerability to addiction. Ann N Y Acad Sci.

[43] Koob GF (2008). A role for brain stress systems in addiction. Neuron.

[44] Thayer JF, Lane RD (2000). A model of neurovisceral integration in emotion regulation and dysregulation. J Affect Disord.

[45] Carroll H, Lustyk MKB (2017). Mindfulness-based relapse prevention for substance use disorders: effects on cardiac vagal control and craving under stress. Mindfulness.

[46] Brewer JA, Sinha R, Chen JA, Michalsen RN, Babuscio TA, Nich C (2009). Mindfulness training and stress reactivity in substance abuse: results from a randomized, controlled stage I pilot study. Subst Abus.

[47] Paz R, Zvielli A, Goldstein P, Bernstein A (2017). Brief mindfulness training de-couples the anxiogenic effects of distress intolerance on reactivity to and recovery from stress among deprived smokers. Behav Res Ther.

[48] Garland EL, Gaylord SA, Boettiger CA, Howard MO (2010). Mindfulness training modifies cognitive, affective, and physiological mechanisms implicated in alcohol dependence: results of a randomized controlled pilot trial. J Psychoactive Drugs.

[49] Goldberg SB, Manley AR, Smith SS, Greeson JM, Russell E, Van Uum S (2014). Hair cortisol as a biomarker of stress in mindfulness training for smokers. J Altern Complement Med.

[50] Marcus MT, Fine PM, Moeller FG, Khan MM, Pitts K, Swank PR (2003). Change in stress levels following mindfulness-based stress reduction in a therapeutic community. Addict Disord Their Treat.

[51] Kober H, Brewer JA, Height KL, Sinha R (2017). Neural stress reactivity relates to smoking outcomes and differentiates between mindfulness and cognitive-behavioral treatments. Neuroimage.

[52] Garland EL, Baker AK, Howard MO (2017). Mindfulness-oriented recovery enhancement reduces opioid attentional bias among prescription opioid-treated chronic pain patients. J Soc Soc Work Res.

[53] Westbrook C, Creswell JD, Tabibnia G, Julson E, Kober H, Tindle HA (2011). Mindful attention reduces neural and self-reported cue-induced craving in smokers. Soc Cogn Affect Neurosci.

[54] Davis JM, Manley AR, Goldberg SB, Smith SS, Jorenby DE (2014). Randomized trial comparing mindfulness training for smokers to a matched control. J Subst Abuse Treat.

[55] Bowen S, Marlatt A (2009). Surfing the urge: brief mindfulness-based intervention for college student smokers. Psychol Addict Behav J Soc Psychol Addict Behav.

[56] Witkiewitz K, Bowen S (2010). Depression, craving, and substance use following a randomized trial of mindfulness-based relapse prevention. J Consult Clin Psychol.

[57] Elwafi HM, Witkiewitz K, Mallik S, Thornhill TA, Brewer JA (2013). Mindfulness training for smoking cessation: moderation of the relationship between craving and cigarette use. Drug Alcohol Depend.

[58] Garland EL, Carter K, Ropes K, Howard MO (2012). Thought suppression, impaired regulation of urges, and addiction-Stroop predict affect-modulated cue-reactivity among alcohol dependent adults. Biol Psychol.

[59] Moss AC, Erskine JA, Albery IP, Allen JR, Georgiou GJ (2015). To suppress, or not to suppress? That is repression: controlling intrusive thoughts in addictive behaviour. Addict Behav.

[60] Bowen S, Witkiewitz K, Dillworth TM, Marlatt GA (2007). The role of thought suppression in the relationship between mindfulness meditation and alcohol use. Addict Behav.

[61] Zgierska A, Rabago D, Chawla N, Kushner K, Koehler R, Marlatt A (2009). Mindfulness meditation for substance use disorders: a systematic review. Subst Abuse.

[62] Katz D, Toner B (2013). A systematic review of gender differences in the effectiveness of mindfulness-based treatments for substance use disorders. Mindfulness.

[63] Chiesa A, Serretti A (2014). Are mindfulness-based interventions effective for substance use disorders? A systematic review of the evidence. Subst Use Misuse.

[64] Li W, Howard MO, Garland EL, McGovern P, Lazar M (2017). Mindfulness treatment for substance misuse: a systematic review and meta-analysis. J Subst Abuse Treat.

[65] Mermelstein LC, Garske JP (2015). A brief mindfulness intervention for college student binge drinkers: a pilot study. Psychol Addict Behav.

[66] Spears CA, Hedeker D, Li L, Wu C, Anderson NK, Houchins SC (2017). Mechanisms underlying mindfulness-based addiction treatment versus cognitive behavioral therapy and usual care for smoking cessation. J Consult Clin Psychol.

[67] Garland EL, Howard MO (2013). Mindfulness-oriented recovery enhancement reduces pain attentional bias in chronic pain patients. Psychother Psychosom.

[68] Brewer JA, Mallik S, Babuscio TA, Nich C, Johnson HE, Deleone CM (2011). Mindfulness training for smoking cessation: results from a randomized controlled trial. Drug Alcohol Depend.

[69] de Dios MA, Herman DS, Britton WB, Hagerty CE, Anderson BJ, Stein MD (2012). Motivational and mindfulness intervention for young adult female marijuana users. J Subst Abuse Treat.

[70] Murphy TJ, Pagano RR, Marlatt GA (1986). Lifestyle modification with heavy alcohol drinkers: effects of aerobic exercise and meditation. Addict Behav.

[71] Onken LS, Carroll KM, Shoham V, Cuthbert BN, Riddle M (2014). Reenvisioning clinical science unifying the discipline to improve the public health. Clin Psychol Sci.

[72] Roos CR, Bowen S, Witkiewitz K (2017). Baseline patterns of substance use disorder severity and depression and anxiety symptoms moderate the efficacy of mindfulness-based relapse prevention. J Consult Clin Psychol.

[73] Witkiewitz K, Lustyk MKB, Bowen S (2013). Retraining the addicted brain: a review of hypothesized neurobiological mechanisms of mindfulness-based relapse prevention. Psychol Addict Behav.

[74] Garland EL, Gaylord SA, Palsson O, Faurot K, Mann JD, Whitehead WE (2012). Therapeutic mechanisms of a mindfulness-based treatment for IBS: effects on visceral sensitivity, catastrophizing, and affective processing of pain sensations. J Behav Med.

[75] Garland EL, Howard MO, Zubieta J-K, Froeliger B (2017). Restructuring hedonic dysregulation in chronic pain and prescription opioid misuse: effects of mindfulness-oriented recovery enhancement on responsiveness to drug cues and natural rewards. Psychother Psychosom.

[76] Van Lutterveld R, Houlihan SD, Pal P, Sacchet MD, McFarlane-Blake C, Patel PR (2017). Source-space EEG neurofeedback links subjective experience with brain activity during effortless awareness meditation. Neuroimage.

[77] Collins LM, Nahum-Shani I, Almirall D (2014). Optimization of behavioral dynamic treatment regimens based on the sequential, multiple assignment, randomized trial (SMART). Clin Trials.

[78] Collins LM, Baker TB, Mermelstein RJ, Piper ME, Jorenby DE, Smith SS (2011). The multiphase optimization strategy for engineering effective tobacco use interventions. Ann Behav Med.

[79] Shonin E, Van Gordon W, Griffiths MD (2014). Are there risks associated with using mindfulness for the treatment of psychopathology?. Clin Pract.

[80] Parsons CE, Crane C, Parsons LJ, Fjorback LO, Kuyken W (2017). Home practice in mindfulness-based cognitive therapy and mindfulness-based stress reduction: a systematic review and meta-analysis of participants’ mindfulness practice and its association with outcomes. Behav Res Ther.

[81] Kabat-Zinn J (1982). An outpatient program in behavioral medicine for chronic pain patients based on the practice of mindfulness meditation: theoretical considerations and preliminary results. Gen Hosp Psychiatry.

[82] Carroll KM (2012). Dissemination of evidence-based practices: how far we’ve come, and how much further we’ve got to go. Addiction.

[83] Simpson DD (2002). A conceptual framework for transferring research to practice. J Subst Abuse Treat.

[84] Woods S. MBSR teacher qualification and certification [Internet]. UCSD Center for Mindfulness. 2018 [cited 2018 Mar 1]. http://mbpti.org/programs/mbsr/mbsr-teacher-certification/.

[85] Chawla N, Collins S, Bowen S, Hsu S, Grow J, Douglass A (2010). The mindfulness-based relapse prevention adherence and competence scale: development, interrater reliability, and validity. Psychother Res.

[86] Dimidjian S, Segal ZV (2015). Prospects for a clinical science of mindfulness-based intervention. Am Psychol.

[87] Jacobson N, Greenley D (2001). What is recovery? A conceptual model and explication. Psychiatr Serv.

[88] Garland EL, Hanley AW, Baker AK, Howard MO (2017). Biobehavioral mechanisms of mindfulness as a treatment for chronic stress: an RDoC perspective. Chronic Stress.

[89] Garland EL, Black DS (2014). Mindfulness for chronic pain and prescription opioid misuse: novel mechanisms and unresolved issues. Subst Use Misuse.

[90] Bateson G (1971). The cybernetics of “self”: a theory of alcoholism. Psychiatry.

[91] Bowen S, Witkiewitz K, Clifasefi SL, Grow J, Chawla N, Hsu SH (2014). Relative efficacy of mindfulness-based relapse prevention, standard relapse prevention, and treatment as usual for substance use disorders: a randomized clinical trial. JAMA Psychiatry.

[92] Spears CA, Hedeker D, Li L, Wu C, Anderson NK, Houchins SC, et al. Mechanisms underlying mindfulness-based addiction treatment versus cognitive behavioral therapy and usual care for smoking cessation. J Consult Clin Psychol [Internet]. 2017 [cited 2017 Aug 7]. https://www.ncbi.nlm.nih.gov/pubmed/28650195. 10.1037/ccp0000229 PMC5662477 28650195

